# Injectable Hydrogel System for Camptothecin Initiated Nanocatalytic Tumor Therapy With High Performance

**DOI:** 10.3389/fonc.2022.904960

**Published:** 2022-06-30

**Authors:** Shuntao Wang, Qi Zhang, Ning Zeng, Pengyuan Qi, Chunyu Huang, Qinqin Huang

**Affiliations:** ^1^Department of Molecular Pathology, The Second Affiliated Hospital of Zhengzhou University, Zhengzhou, China; ^2^Department of Breast and Thyroid Surgery, Union Hospital, Tongji Medical College, Huazhong University of Science and Technology, Wuhan, China; ^3^Department of Plastic Surgery, Tongji Hospital, Tongji Medical College, Huazhong University of Science and Technology, Wuhan, China; ^4^The Institute for Advanced Studies, Wuhan University, Wuhan, China; ^5^Department of Radiation and Medical Oncology, Hubei Key Laboratory of Tumor Biological Behaviors, Hubei Cancer Clinical Study Center, Zhongnan Hospital of Wuhan University, Wuhan, China

**Keywords:** hydrogel, pyrite nanozyme, camptothecin, photothermal therapy, catalytic treatment

## Abstract

Single photothermal therapy (PTT) has many limitations in tumor treatments. Multifunctional nanomaterials can cooperate with PTT to achieve profound tumor killing performance. Herein, we encapsulated chemotherapeutic drug camptothecin (CPT) and pyrite (FeS_2_) with dual enzyme activity (glutathione oxidase (GSH-OXD) and peroxidase (POD) activities) into an injectable hydrogel to form a CFH system, which can improve the level of intratumoral oxidative stress, and simultaneously realize FeS_2_-mediated PTT and nanozymes catalytic treatment. After laser irradiation, the hydrogel gradually heats up and softens under the photothermal agent FeS_2_. The CPT then released from CFH to tumor microenvironment (TME), thereby enhancing the H_2_O_2_ level. As a result, FeS_2_ can catalyze H_2_O_2_ to produce **·**OH, and cooperate with high temperature to achieve high-efficiency tumor therapy. It is worth noting that FeS_2_ can also deplete excess glutathione (GSH) in the cellular level, further amplifying oxidative stress. Both *in vivo* and *in vitro* experiments show that our CFH exhibits good tumor-specific cytotoxicity. The CFH we developed provides new insights for tumor treatment.

## Introduction

As the main cancer treatment methods in the world, surgical resection, chemotherapy and radiotherapy have achieved certain results, but the side effects and other limitations after treatment limit their effects (1–4). In recent years, photothermal therapy has been proposed for non-invasive treatment of tumors (5, 6). Photothermal therapy uses nanomaterials with high near-infrared absorption characteristics to generate heat under laser irradiation and subsequently induce tumor ablation (7). Since near-infrared light penetrates biological bodies more easily than ultraviolet light and visible light, near-infrared light is widely used in PTT (8). In order to enhance the effect of penetration therapy, researchers have designed a variety of new nanomaterials, including metal nanoparticles, organic polymer nanoparticles, carbon-based nanoparticles, etc. to improve the light-to-heat conversion ability (9, 10). Photothermal nanomaterials must have high photothermal conversion efficiency, high surface modification activity, high bio-histocompatibility, and low toxicity (11, 12). PTT is safer than radiotherapy and many molecular drugs (11–13). Although certain effects have been achieved, due to the complexity of the tumor microenvironment, a single PTT is difficult to completely eradicate the tumor tissue (14, 15). Therefore, it is necessary to develop multifunctional nanomaterials, which can not only realize PTT, but also achieve better therapeutic effects in response to the tumor microenvironment.

Nanozymes with mimic biological enzyme activity can be combined with photothermal therapy to inhibit the growth of malignant tumors, as nanozymes can affect biological activity at the molecular or cellular level according to the particularity of the tumor microenvironment (16, 17). For example, Fe3O4 nanoparticles can catalyze endogenous tumor hydrogen peroxide (H_2_O_2_) to produce hydroxyl radical (•OH), which realizes tumor catalysis treatment, simultaneously, Fe3O4 nanoparticles have a good photothermal effect (18). Furthermore, Cui and his team synthesized a novel type of FePPy nanozyme (19). Under laser irradiation, FePPy nanoparticles located in tumor tissue can trigger photothermal transformation and enhance ferroptosis by enhancing fenton reaction (19). Both in vito and in vivo experiments showed that FePPy combined with photothermal treatment has observed significant tumor suppression. However, tumor microenvironment (TME) often contains high levels of glutathione (GSH) (10 mM in TME) (20), and GSH could react with •OH, thereby reducing the corresponding treatment efficiency. As a novel type of photothermal nanomaterial, pyrite (FeS_2_) nanozymes can not only respond to near-infrared light and generate a lot of heat to destroy tumor tissues, but also have multiple nanozyme activities (21). FeS_2_ nanozyme can oxidize GSH to oxidized glutathione (GSSG), that is, it has similar activity with glutathione oxidase (GSH-OXD) (22). At the same time, as pyrite has a very high affinity for the substrate H_2_O_2_, it can also effectively catalyze the limited H_2_O_2_ in the TME and produce more cytotoxic •OH for tumor treatment. However, due to the limited content of endogenous H_2_O_2_ in tumor, it is difficult to maintain the catalytic effect of nanozyme for a long time (16, 23). Therefore, in order to improve the therapeutic effect of FeS_2_-based tumor catalysis and photothermal treatment, a self-produced H_2_O_2_ system is urgently needed.

In addition, the traditional methods of delivering nanomaterials to tumor sites are through oral or vein injection (13, 24), which will lead to a series of problems such as premature release of the carrier, missing the optimal treatment time of the carrier, and long-term toxic and side effects caused by the carrier’s residence time in the body and other problems (25–27). Local therapeutic drug delivery is an attractive alternative to systemic intravenous drug delivery, enabling researchers to achieve sustained and precise release of nanomaterials without any risk of off-target toxicity (28, 29). NIR light-responsive hydrogel is a satisfactory and controllable drug delivery platform (30). The hydrogel is gradually solidified after being injected into the tumor tissue. It can be used as a long-term reservoir. Zhu et al. designed an injectable nano-enzyme hydrogel as the storage and controlled release of AIEgen to achieve effective tumor treatment (31). Given these findings, we hypothesized that the use of hydrogels to deliver FeS_2_ to TME would improve photothermal efficacy.

**Scheme 1:**
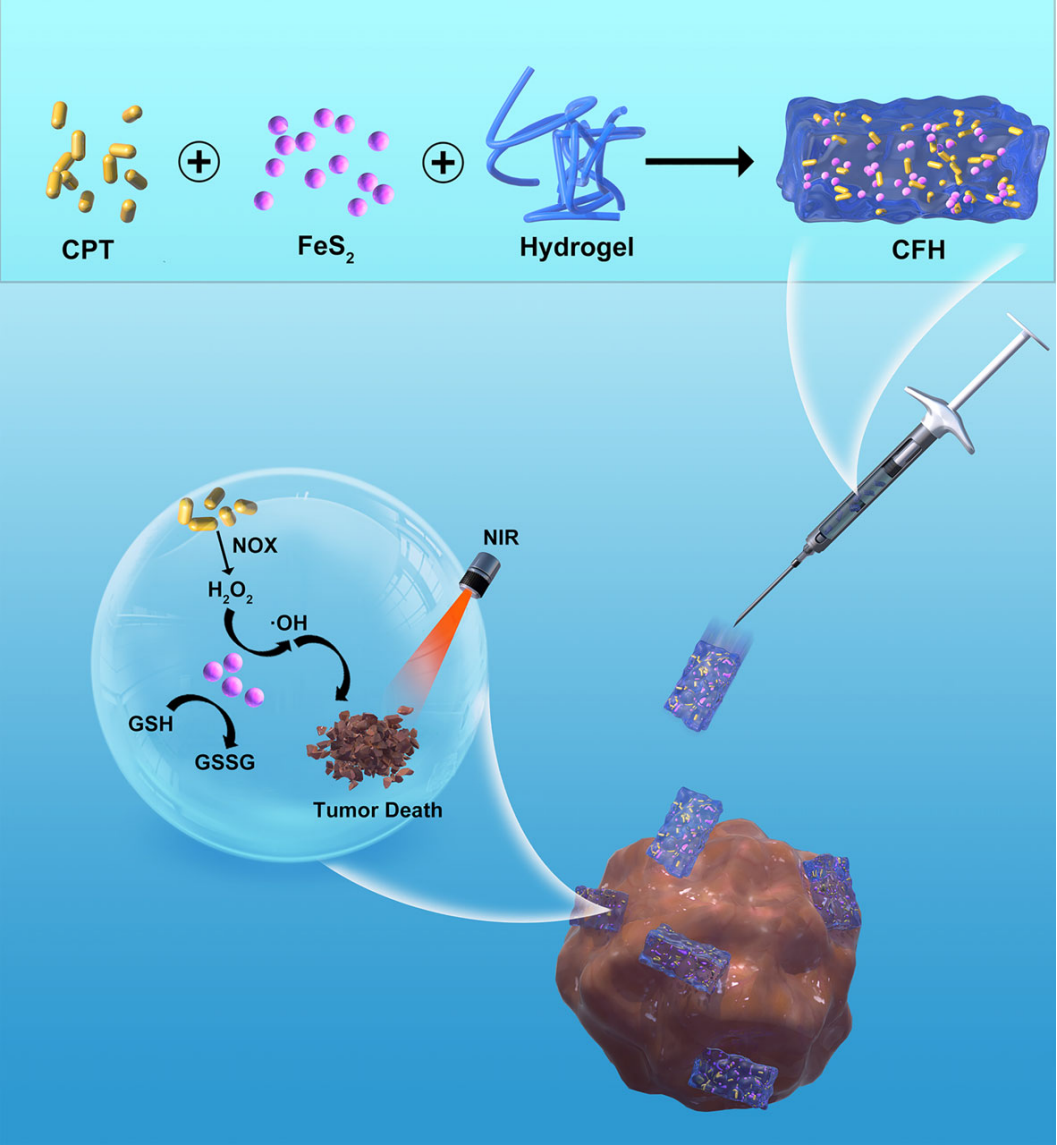
Schematic illustration of injectable hydrogel system for camptothecin initiated nano-catalytic tumor therapy with high performance.

Here, we design an intratumoral administration method of an injectable composite hydrogel (CFH) (**Scheme 1**) containing the chemotherapeutic drug camptothecin (CPT) and FeS_2_ nanoparticles for the combined use of photothermal and nanozyme-catalyzed therapy, CFH can be used to regulate the release of FeS_2_ and CPT under light irradiation. Once irradiated by NIR irradiation, FeS_2_ can realize the conversion of light energy to heat energy, and at the same time realize local tumor ablation and hydrogel dissolution, thereby releasing the CPT in CFH. CPT will spread to the local TME to kill tumor cells, and increase the production of H_2_O_2_ by activating nicotinamide adenine dinucleotide phosphate (NADPH) oxidase (NOX) (32), so that FeS_2_ can produce enough •OH to further enhance the death of tumor cells. Since CFH can stay at the tumor site for a long time after injection, we could change the laser parameters to adjust the release of nanomaterials and CPT to keep it within its treatment window. Both in vivo and in vitro experiments show that our CFH drug delivery platform achieves good anti-tumor efficacy of FeS_2_-based photothermal therapy without systemic toxicity. Therefore, the CFH drug delivery platform can not only expand the application field of FeS_2_ nanozymes, but it is also the first system that enhances FeS_2_-based cancer treatment by rationally designing functional nanocarriers with H_2_O_2_ self-supply and tumor specificity.

## Results and Discussion

**Figure 1:**
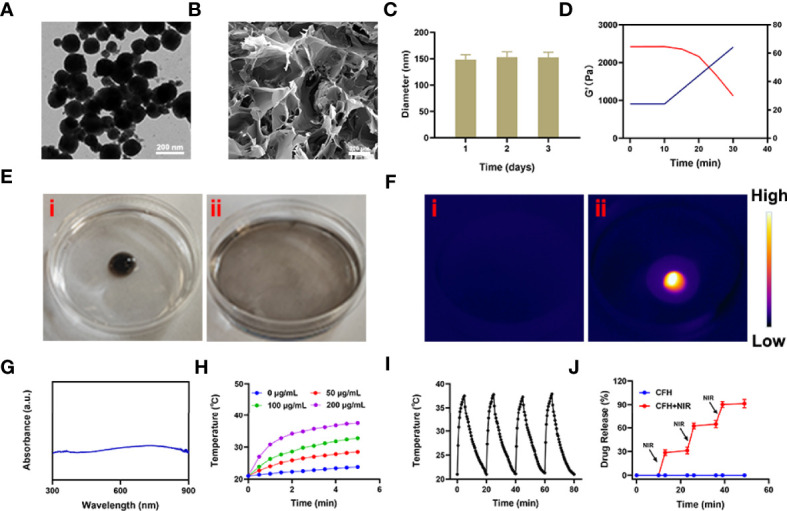
**(A)** TEM image of FeS_2_ nanozymes. **(B)** SEM image of hydrogel. **(C)** Statistical graph of measured diameter size of FeS_2_. **(D)** Rheological and temperature curves (red and blue, respectively) for the prepared CFH under conditions that simulate an exposure to 0.5 W/cm^2^ 808 nm laser irradiation. **(E)** The morphology of the prepared CFH before (i) and after (ii) 0.5 W/cm^2^ 808 nm laser irradiation for 10 min. **(F)** The infrared thermal image of the prepared CFH before (i) and after (ii) irradiation. **(G)** UV–vis spectra of FeS_2_. **(H)** Temperature changes of FeS_2_ at different concentrations under a 5 min irradiation from an 808 nm laser at 0.5 W/cm^2^. **(I)** Temperature variations of a FeS_2_ solution over four cycles of heating and natural cooling. **(J)** *In vitro* CFH CPT release profile in the presence and absence of 808 nm laser irradiation.

We mixed the low melting point agarose hydrogel with FeS_2_ nanozyme and CPT, and kept stirring at 60 degrees to prepare the CFH system. **Figure 1A** shows the transmission electron microscope (TEM) of FeS_2_. The particle size of FeS_2_ we prepared is about 150 nm. As shown in **Figure 1B**, the scanning electron microscope (SEM) showed that the hydrogel presented a three-dimensional complex network structure. We first verified the stability of FeS_2_ and measured the Zeta potential and particle size of FeS_2_ for three days. As shown in **Figure 1C**, the particle diameters are about 148.2 ± 5.6 nm, 153 ± 4.7 nm and 152.6 ± 6.2 nm respectively. There is almost no change in the particle size, and the zeta potential also tends to be stable (**Figure S1**). The rheological measurement results of CFH at different temperatures show that as the temperature rises, CFH will gradually soften, and the storage modulus of CFH will continue to decrease (**Figure 1D**). This result is consistent with the rheological properties of the hydrogel (33), as shown in **Figure 1D**. After CFH is prepared, it will be very stable when stored at room temperature. Under continuous laser irradiation, CFH will gradually release FeS_2_ in it, and the solution will become turbid (**Figure 1E**). Infrared thermal images verify the temperature difference before and after irradiation (**Figure 1F**). FeS_2_ can promote the conversion of light energy to heat energy, leading to an increase in temperature. The UV-Vis absorption spectrum verifies FeS_2_ has an absorption value at 808nm, showing a wide absorption region in the near infrared region. (**Figure 1G**), which is also the factor that it can respond to 808 nm laser irradiation. A good photothermal agent is conducive to photothermal treatment. We prepared FeS_2_ solutions of different concentrations (0, 50, 100, 200 μg/mL) and utilized 0.5 W/cm2 laser for processing (**Figure 1H**). The results showed that the solution heating effect was positively correlated with the material concentration, and the 200 μg/mL FeS_2_ solution could rise by about 16.5 degrees under 5 minutes of laser irradiation. One of the most important factors for evaluating photothermal agent (PTA) is the photothermal stability (34). Next, we continue to use the 808 nm near-infrared laser to repeatedly heat the FeS_2_ solution, and close the switch after 5 min to allow the FeS_2_ to naturally cool to ambient temperature (**Figure S2**). We performed four heating and cooling cycles (**Figure 1I**). The temperature curve shows that the peak temperature of FeS_2_ has a small change, and the cooling trend is similar, which also shows photothermal stability of FeS_2_ nanoparticles. As shown in **Figure 1J**, once the CFH is exposed to laser radiation, it will achieve a photothermal response drug release. After the temperature rises, the CPT is gradually released. After the irradiation is stopped, the hydrogel continues to restore the colloidal state. This result shows that the CFH prepared by us can achieve good storage and release of the CPT.

**Figure 2:**
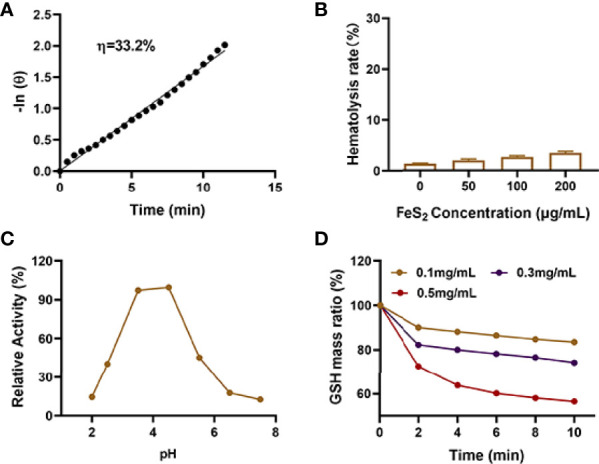
**(A)** Calculation of the time constant for heat transfer using a linear regression of the cooling profile. **(B)** Hemolysis ratio of FeS_2_ at different FeS_2_ concentrations. **(C)** pH-dependent POD activity of FeS_2_. **(D)** Time-dependent reduction of GSH after incubating with the indicated concentrations of FeS_2_.

The photothermal conversion efficiency (η) of FeS_2_ was 33.2% (**Figure 2A)** in our experiments. Only nanomaterials with good biocompatibility can be applied for subsequent biological experiments (35). As shown in **Figure 2B**, FeS_2_ is stable in blood, and even 200 μg/mL FeS_2_ would not cause hemolysis. We continued to explore the effect of pH on the POD-like activity of FeS_2_. The result showed FeS_2_ has the strongest POD enzyme activity under acidic conditions with a pH of 4.5, while the enzyme activity is lower under normal neutral conditions (**Figure 2C)**. TME is weakly acidic, which promotes POD activity of FeS_2_ enzyme. We applied 5,5′-dithiobis (2-nitrobenzoic acid) (DTNB) as a probe to analyze the GSH-OXD enzyme activity of FeS_2_ at different concentrations. The co-incubation of FeS_2_ and GSH will lead to a significant decrease in GSH content (**Figure 2D**), and it has a positive correlation curve with time and concentration. These results verified the good dual enzyme activity of FeS_2_, and FeS_2_ can also be used as an ideal photothermal agent.

**Figure 3:**
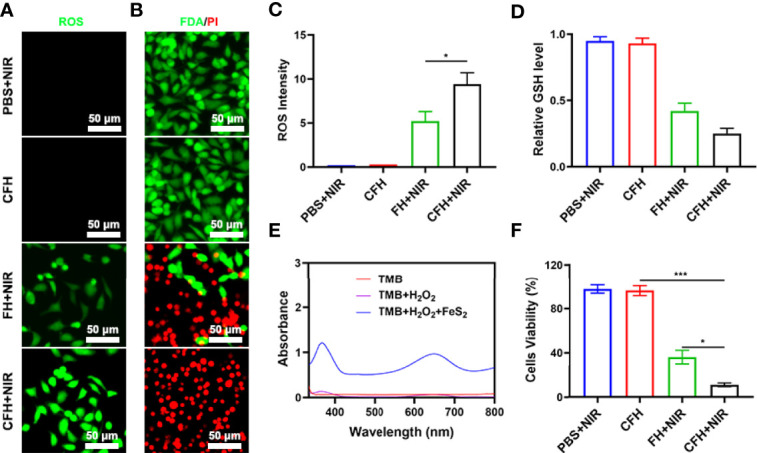
**(A)** DCFH-DA fluorescence image under the indicated treatments. **(B)** Fluorescence images of CT26 cells stained with fluorescein diacetate (FDA) (live cells, green fluorescence) and propidium iodide (PI) (dead cells, red fluorescence) after incubation with different formulations. **(C)** DCFH-DA fluorescence intensity after the indicated treatments. **(D)** The impact of FeS_2_ on the intracellular level of GSH was estimated using a GSH assay kit (n = 5). **(E)** UV − vis absorbance spectra and color changes of TMB in different reaction systems. **(F)** MTT assays were conducted using CT26 cells treated with different formulations. *P < 0.05, ***P < 0.005; Student’s t-test.

Due to the good nanozyme activity of FeS_2_, we verified the ability of FeS_2_ to generate ROS in vitro. Although the content of H_2_O_2_ in tumor cells (up to 0.1-1 mM) is higher than that in normal cells, its content is still limited and is not conducive to continuous tumor treatment (36). Our CFH system contains CPT that can increase H_2_O_2_ and can cooperate with FeS_2_ to produce reactive oxygen (ROS) (**Figure S3**). As shown in **Figure 3A**, the dichlorofluorescein diacetate (DCFH-DA) probe was used to detect the ability of CFH to produce ROS. There was almost no green fluorescence of ROS in the control group, and CFH group. We prepared a hydrogel containing only FeS_2_ (FH) for exploratory experiments to verify the ability of CPT to produce H_2_O_2_. As shown in **Figures 3A, C**, the FH combined with NIR group produced moderate fluorescence effect and CFH + NIR motivate the strongest green fluorescence. We then used a live dead cell staining kit (Fluorescein diacetate and propidium iodide are living and dying cell fluorescent tracer probe) to explore the tumor cell killing effect of CFH (**Figure 3B**). Under laser irradiation, the hydrogel containing only FeS_2_ can realize the conversion of light energy to heat energy, and induce the death of some tumor cells, with moderate Cell killing effect. The CFH + NIR group achieved the best therapeutic effect. The content of GSH is high in tumors, and GSH is common in various types of cancer tissues. GSH is a highly reducing substance, which generally exists in a simplified form in cells, and can react with oxidative reaction substances (37). GSH reacts with reactive oxygen species to be oxidized and subsequently forms GSSG, thereby inhibiting the anti-tumor effect based on ROS. Based on the depletion of glutathione, free radicals destroy the redox balance of cells, cause oxidative stress, and ultimately lead to cell apoptosis (38). We have verified the effect of CFH in cooperating with NIR irradiation to consume GSH (**Figure 3D**). The results show that FeS_2_ can well reduce intracellular GSH levels, and CPT can also produce a certain amount of H_2_O_2_ to promote GSH consumption, thereby further improving FeS_2_ nanozymes catalytic therapeutic effect. **Figure 3E** also exhibit good POD-like activity of FeS_2_. We use the MTT assay to further verify the therapeutic effect of CFH + NIR. The cell viability of the control group and the CFH group was very high, while the cell viability of the FH+NIR group was about 36.5%, while the cell viability of the CFH+NIR group was the lowest, only 10.8% (**Figure 3F**). This also shows that CFH has good anti-tumor ability in vitro.

**Figure 4:**
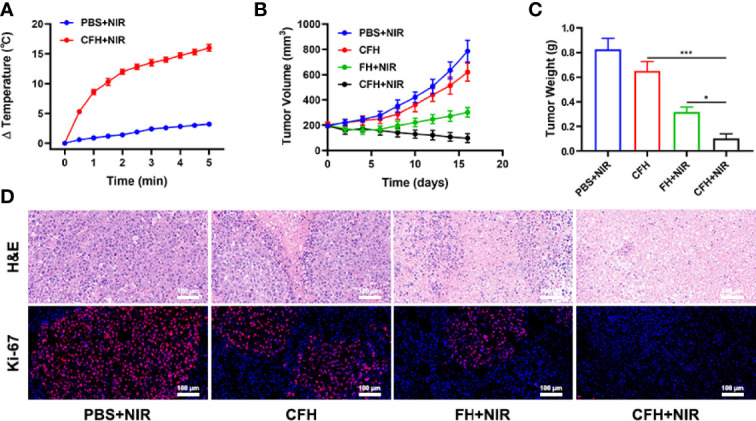
**(A)** Temperature increases in mice implanted with CT26 tumors following 808 nm laser irradiation (0.5 W/cm^2^) for 5 min in the indicated treatment groups. **(B)** Tumor volume change over time in groups treated as indicated. **(C)** Average tumor weight values associated with the indicated treatments. **(D)** H&E and Ki-67 stained tumor sections from the indicated treatment groups. *P < 0.05, ***P < 0.005; Student’s t-test.

In view of the fact that FeS_2_ exhibits better photothermal performance in vitro, we continue to explore the photothermal effect of CFH in vivo. As shown in **Figure 4A**, the control group achieved a temperature rise of less than 5°C after five minutes under the 808 nm laser irradiation. CFH + NIR, on the other hand, resulted in a temperature rise of about 16.4°C (**Figure 4A**). It shows that CFH is suitable for photothermal therapy, and the low power density we utilized is also conducive to reducing the damage of hyperthermia to surrounding healthy tissues. The heat resistance of tumor tissue is lower than that of normal cells, causing active substances such as proteins in tumor cells to be destroyed at high temperature (42-47°C), and then induce cell apoptosis (39). To explore the synergistic in vivo antitumor ability of CFH and NIR in mice with CT26 tumors, BALB/c mice were injected subcutaneously with 1 × 106 CT26 cells to assess the main effect of CFH. The mice were treated after reaching the primary tumor volume to about 200 mm3. Tumor-bearing mice were randomly arranged into 4 groups (5 mice per group) (1): PBS + NIR (2); CFH (3); FH + NIR and (4) CFH + NIR. The FeS_2_ concentration was 20 mg/kg in group 2, 3 and 4. Then, mice in group 1, 3 and 4 were exposed to 808 nm laser radiation (0.5 W/cm2) for 10 min. Mice body weight was monitored every 2 days. During the continuous treatment cycle, the tumor volumes in the control group (PBS + NIR) increased significantly, while the CFH group achieved only a negligible tumor suppression effect. FH + NIR group indicates a better therapeutic effect. Although FH combined with laser has a certain tumor ablation effect, the intracellular H_2_O_2_ content limits the further catalytic therapeutic effect of FeS_2_. The treatment group of CFH combined with NIR showed the best tumor suppression curve (**Figure 4B**). This is because after laser irradiation, the chemotherapy drug CPT can not only promote tumor cell apoptosis, but also increase H_2_O_2_ levels and further enhance FeS_2_-mediated ·OH production, while FeS_2_ can also deplete intracellular GSH to achieve oxidative stress damage. The tumor weight in mice after treatment was consistent with the results (**Figure 4C**). The agarose hydrogel was safe and non-toxic, and there were no abnormal changes in the body weight of the mice throughout the treatment cycle, indicating that our treatment regimen was safe (**Figure S4**). Furthermore, staining of Ki-67 was conducted and observed to be obviously decreased after the treatment of CFH combined with laser irradiation group. The hematoxylin and eosin (H&E) staining results demonstrated that the structure of solid tumor tissue was destroyed, and many tumor cells are necrotic after treatments of CFH synergistic with NIR and the cells in the tissue (**Figure 4D**) were contracted with disappeared nuclei. These results suggested that our CFH can cooperate with laser irradiation to realize tumor treatment. The potential in vivo toxicity of nanomaterials has limited their clinical biological applications (40, 41). After the treatment, all mice were euthanized, and their blood and major organs were collected for further analysis. We obtain the vital organs (Heart, liver, spleen, lung and kidney) of the mice for histopathological analysis, and the results showed that our treatment system was safe and non-toxic, and the liver and kidney indexes were also normal. In summary, in vivo studies have shown that our CFH can not only achieves the optimal tumor killing effect, but also has a good in vivo safety.

**Figure 5:**
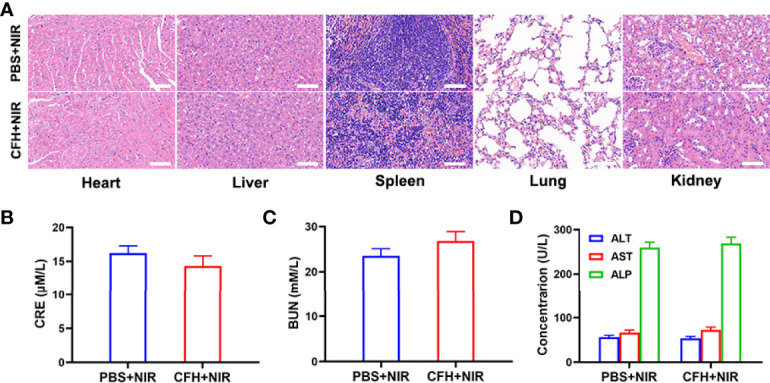
**(A)** Histopathological analysis results (H&E stained images) of the major organs, heart, lung, liver, kidneys, and spleen, of mice that were exposed to different treatments 16 days post-injection. Scale bars: 100 μm. **(B)** Liver function markers: CRE, **(C)** BUN and **(D)** ALT, AST and ALP after various treatments.

## Conclusion

In conclusion, we prepared a CFH system for tumor ablation using agarose hydrogel with high safety, combined with chemotherapeutic drug CPT and FeS_2_ nanozyme with both GSH-OXD and POD activities. FeS_2_ can guide the conversion of light energy to heat energy and promote the release of CPT. The hydrogen peroxide produced can further enhance the therapeutic effect of FeS_2_. *In vivo* experiments have shown that CFH combined with NIR can destroy tumor tissue and inhibit tumor growth with good safety. Tumor tissue has a unique pathological environment, including dense extracellular matrix (ECM) and abnormal vascular system. Hydrogels can help FeS_2_ reach tumor tissue and achieve the good anti-tumor effect. This platform has great potential for treating solid tumors.

## Data Availability Statement

The original contributions presented in the study are included in the article/**Supplementary Material**. Further inquiries can be directed to the corresponding author.

## Ethics Statement

The animal experiments were carried out according to the protocol approved by the Ministry of Health in People’s Republic of PR China and were approved by the Administrative Committee on Animal Research of the Wuhan University.

## Author Contributions

SW: methodology, validation, formal analysis, roles, and data curation. QZ: investigation, formal analysis, formal analysis, and writing – original draft. NZ: conceptualization and project administration. PQ: writing – review and editing. CH: conceptualization and writing – original draft. QH: funding acquisition, writing – original draft, writing – review and editing, and resources. All authors contributed to the article and approved the submitted version.

## Funding

This work was supported by National Natural Science Foundation of China (31800085).

## Conflict of Interest

The authors declare that the research was conducted in the absence of any commercial or financial relationships that could be construed as a potential conflict of interest.

## Publisher’s Note

All claims expressed in this article are solely those of the authors and do not necessarily represent those of their affiliated organizations, or those of the publisher, the editors and the reviewers. Any product that may be evaluated in this article, or claim that may be made by its manufacturer, is not guaranteed or endorsed by the publisher.
