# Supplementary material for: Injectable Hydrogel System for Camptothecin Initiated Nanocatalytic Tumor Therapy With High Performance
Source: Front Oncol. 2022 Jun 30;12:904960. doi: 10.3389/fonc.2022.904960 (PMC9280668; doi:10.3389/fonc.2022.904960)
Supplement: Supplementary file 1 [file DataSheet_1.docx]

**Materials.**

Ethylene glycol (EG), iron (III) chloride hexahydrate (FeCl_3_·6H2O, 99%) anhydrous sodium acetate (NaAc, 99%) were purchased from Sigma-Aldrich. NaOH, ethylene glycol and ammonium hydroxide solution were purchased from Sinopharm Chemical Reagent Co., Ltd. Agarose was purchased from Yare Shanghai. The reduced GSH assay kit was purchased from Nanjing Jiancheng Bioengineering Institute. Sulfur powder (S, 99%) was obtained from Shanghai Rongtai Pharmatech Co. Gibco RPMI 1640 medium were purchased from Invitrogen (Shanghai) Trading Co. The other reagents used in this work were purchased from Sinopharm Chemical Reagent (China) and Aladdin-Reagent (China).

**Preparation and characterization of pyrite nanozymes (FeS_2_)[**[**1**](#_ENREF_1)**].**

The pyrite nanozymes with a size of 148 nm were prepared by a one-pot solvothermal method. In a typical synthesis, 0.7 g of PVP was dispersed into 30 mL of EG solution, and then 0.5 g of FeCl_3_·6H_2_O was added under constant magnetic stirring. Next, 3.6 g of NaAc was added into above mixture under vigorous stirring. After that, 0.4 g of S powder was added, and the resultant mixture was ultrasonicated for 1 h to form a homogeneous dispersion. Then, the reaction mixture was transferred into a 40 mL Teflon-lined stainless-steel autoclave, which was sealed and maintained at 473 K for 12 h. After the reaction was cooled to room temperature naturally, the resultant black precipitates were collected by centrifugation at 10 000 rpm for 10 min. Then, the collected black products were washed with CHCl_3_ to remove the excess S and using absolute alcohol and ultrapure water several times to remove impurities. After centrifugation, the products were dried in a vacuum lyophilizer overnight for further characterization. The UV-vis absorbance spectra of FeS_2_ were recorded via UV-vis spectrophotometry Lambda 35 (Perkin-Elmer). The morphology structures of FeS_2_ were detected by the TEM (JEOL-2100). The size and zeta potential of FeS_2_ were measured by dynamic light scattering (DLS).

**POD-like Activity.**

The POD-like activity of FeS_2_ was assessed using TMB as the substrate in the presence of H_2_O_2_. To determine whether pyrite nanozymes show POD-like activity, 0.1mg of FeS_2_ was added into PBS buffer solution (pH 6.5) containing 1 μL of TMB (20 mg/mL in DMSO) and 5 μL of H_2_O_2_ (10 mM). The UV-vis absorbance spectra of oxidized TMB were recorded via UV-vis spectrophotometry Lambda 35 (Perkin-Elmer). The POD activity test method under different pH values is similar to the above.

**Preparation and characterization of FeS_2_@CPT hydrogels (CFH)**

The general protocol for the hydrogel preparation is as follows. The prepared FeS_2_ (3mg in PBS) and CPT (500 μg in DMSO) were mixed into 3% agarose solution to form CFH.

**Rheological Test**

Rheology experiments were performed on an Anton Paar rheometer. Hydrogel samples of different temperatures were prepared and gently placed on the middle of a 15 mm diameter parallel plate with a proper gap. Dynamic oscillatory frequency sweep measurements were conducted at a 1% strain amplitude. To prevent the evaporation of water, a lid was prepared on the top.

**Photothermal Conversion Efficiency**

An 808 nm NIR laser (Changchun New Industries Tech.Co., Ltd., Changchun, China) with irradiation powers was used to stimulate the concentrations of FeS_2_ (200 ug/mL) in an aqueous medium. The photothermal images of the FeS_2_-based suspensions during laser irradiation were recorded every 30 s using an infrared thermal imaging system. The NIR laser source was equipped with a 4 mm diameter laser module with an adjustable power. The photothermal conversion efficiency was calculated using the following equation[[2](#_ENREF_2)]:


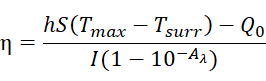


where h is the heat transfer coefficient, S is the surface of the container, T_max_ and T_surr_ are the equilibrium temperature and ambient temperature, respectively. Q_0_ is the heat associated with the light absorbance of the solvent, A_λ_ is the absorbance of FeS_2_at 808 nm, and I is the laser power density. According to the above equation, the η value of FeS_2_ was determined to be about 33.2%.

**CPT release study**

The in vitro CPT release profile from CFH was carried out. 1mL of CFH containing 20 μg CPT was added into culture dish. To investigate the stimuli effect of laser irradiation on the release behavior, the release experiment of CPT was initially performed with or without 0.5 W/cm^2^ 808 nm laser irradiation for 3 min. At appropriate time point, 100 μL of different samples were collected, and an UV-vis spectrophotometer was used to monitor the released CPT content.

**Cell line.** CT26 mouse colon cancer cell line was obtained from the Cell Bank of the Chinese Academy of Sciences and incubated in RPMI-1640 medium supplemented with 10% FBS in a humidified atmosphere at 37 °C.

**Detection of Intracellular GSH.**

The commercially available GSH assay kit was used to detect the depletion of GSH. CT26 cells were seeded in 96-well plates at a density of 5 × 10^3^ cells per well and incubated for 12 h under normoxia condition. Afterwards, cells were incubated for 4 different groups: (1) PBS+NIR; (2) CFH; (3) FH+NIR; (4) CFH + NIR. The CPT concentration was 5 μg/mL in group 2, and 4. Then, cells in group 1, 3 and 4 were exposed to 808 nm laser radiation (0.5 W/cm^2^) for 5 min. After 12 hours of incubation, the GSH content was measured by employing a commercial colorimetric GSH assay kit. The assay was carried out according to the manufacturer’s instructions. The consumption of GSH with different concentrations of FeS_2_ was also tested in a similar way.

**Hemolysis assay**

A hemolysis assay was carried out to evaluate the cytotoxicity of FeS_2_ in vitro. Rabbit heart blood (5 mL) was stabilized by ethylenediamine tetraacetic acid (EDTA) (0.2 mL), an anticoagulant agent. Then red blood cells (RBCs) were obtained from the rabbit heart blood by centrifugation and washing with PBS (2%). 0.5 mL of the RBC solution was then mixed with 0.5 mL FeS_2_ PBS solution at different concentrations (50, 100 and 200 μg/mL FeS_2_). Water and PBS were used as the positive and negative controls, respectively. All samples were mixed gently and kept at room temperature for 3 h. The absorbance of each supernatant, obtained by centrifugation, was measured at 570 nm on a UV–vis photospectrometer. The hemolysis ratio was calculated by using the formula: hemolysis ratio = (sample absorbance − negative control absorbance) / (positive control absorbance − negative control absorbance) × 100%.

**NADPH oxidase activity**

The activity of NADPH oxidase was determined in membrane fractions (50 μg of protein) incubated with 1 mM EGTA and 5 μM lucigenin in phosphate buffer, pH 7.0. The assay was initiated by the addition of 50 μM NADPH to the incubation mixture. Samples were counted immediately using a tabletop luminometer with sampling time every 6 s. Samples were counted over a period of 5 min, and the fluorescence values were recorded for over 2 min of stable readings and averaged for that sample.

***In vitro* anti-cancer effect of CFH**

The anti-tumor effect was measured by MTT assay. CT26 cells were seeded in 96-well plates at a density of 5 × 10^3^ cells per well and incubated for 12 h under normoxia condition. Afterwards, cells were incubated for 4 different groups: (1) PBS+NIR; (2) CFH; (3) FH+NIR; (4) CFH + NIR. The CPT concentration was 5 μg/mL in group 2, and 4. Then, cells in group 1, 3 and 4 were exposed to 808 nm laser radiation (0.5 W/cm^2^) for 5 min. At the end of the incubation, 5 mg/mL MTT PBS solution was added, and the plate was incubated for another 4 h. Finally, the absorbance values of the cells were determined by using a microplate reader (Emax Precision, USA) at 570 nm. The background absorbance of the well plate was measured and subtracted. The cytotoxicity was calculated by dividing the optical density (OD) values of treated groups (T) by the OD values of the control (C) (T/C × 100%).

To further visualize the cell phototoxicity of each group, cells were incubated for 4 different groups: (1) PBS+NIR; (2) CFH; (3) FH+NIR; (4) CFH + NIR. The CPT concentration was 5 μg/mL in group 2, and 4. Then, cells in group 1, 3 and 4 were exposed to 808 nm laser radiation (0.5 W/cm^2^) for 5 min. Then, all cells were washed with PBS, treated with FDA and PI according to the manufacturer’s protocol, and detected under a fluorescent microscope (IX81, Olympus, Japan).

***In vitro* ROS generation**

ROS generation was also assessed *in vitro* on CT26 cells. Briefly, cells were incubated for 4 different groups: (1) PBS+NIR; (2) CFH; (3) FH+NIR; (4) CFH + NIR. The CPT concentration was 5 μg/mL in group 2, and 4. Then, DCFH-DA was used to detect different ROS level. Then, cells in group 1, 3 and 4 were exposed to 808 nm laser radiation (0.5 W/cm^2^) for 5 min and detected under a fluorescent microscope (IX81, Olympus, Japan).

**Animal tumor models**

Female BALB/c mice aged 4-5 week were purchased from Vital River Company (Beijing, China). 100 μL of CT26 cell suspension (1×10^6^ cells per mL) were subcutaneous injected into each mouse to establish the tumor models. The animal experiments were carried out according to the protocol approved by the Ministry of Health in People’s Republic of PR China and were approved by the Administrative Committee on Animal Research of the Wuhan University.

***In vivo* infrared thermography**

To monitor the in vivo photothermal effect, CFH (SAzyme: 2 mg/kg, CPT: 0.5 mg/kg) was intratumorally injected into the tumor-bearing mice, and then the tumors suffered from 0.5 W/cm^2^ irradiation for 3 min at 1 h post-injection. PBS injection used as control group. Meanwhile, the temperature at the tumor was monitored using an infrared camera (Fotric 225).

***In vivo* antitumor study.**

To investigate the abscopal effect of the CFH, Balb/c mice were subcutaneous injected with 1 × 10^6^ CT26 cells into the right flank (primary tumors) and 2 × 10^5^ CT26 cells into the left flank (abscopal tumors), respectively. The mice were grouped and treated when the primary tumor volume reached 200 mm^3^. Tumor bearing mice were divided randomly into 4 groups (each group included 5 mice): (1) PBS+NIR; (2) CFH; (3) FH+NIR; (4) CFH + NIR. The FeS_2_ concentration was 20 mg/kg in group 2, 3 and 4. Then, mice in group 1, 3 and 4 were exposed to 808 nm laser radiation (0.5 W/cm^2^) for 10 min. Mice body weight was monitored every 2 days. After 16 days, all the mice were sacrificed. The blood samples from these mice (≈1 mL) were collected for blood biochemistry analysis. Five main organs (heart, liver, spleen, lung and kidney) and tumors of all mice were harvested, washed with PBS, and fixed with paraformaldehyde for histology analysis. And the tumor tissues were imaged and weighed, and fixed in 4% neutral buffered formalin, processed routinely into paraffin, and sectioned at 4 μm. Then the sections were stained with Terminal deoxynucleotidyl transferase-mediated deoxyuridinetriphosphate nick end labeling (TUNEL), Ki-67 and H&E and finally examined by using an optical microscope (BX51, Olympus, Japan).

**Statistical analysis**Data analyses were conducted using the GraphPad Prism 5.0 software. Significance between every two groups was calculated by the Student’s *t*-test. *P < 0.01, **P < 0.005, ***P < 0.001.


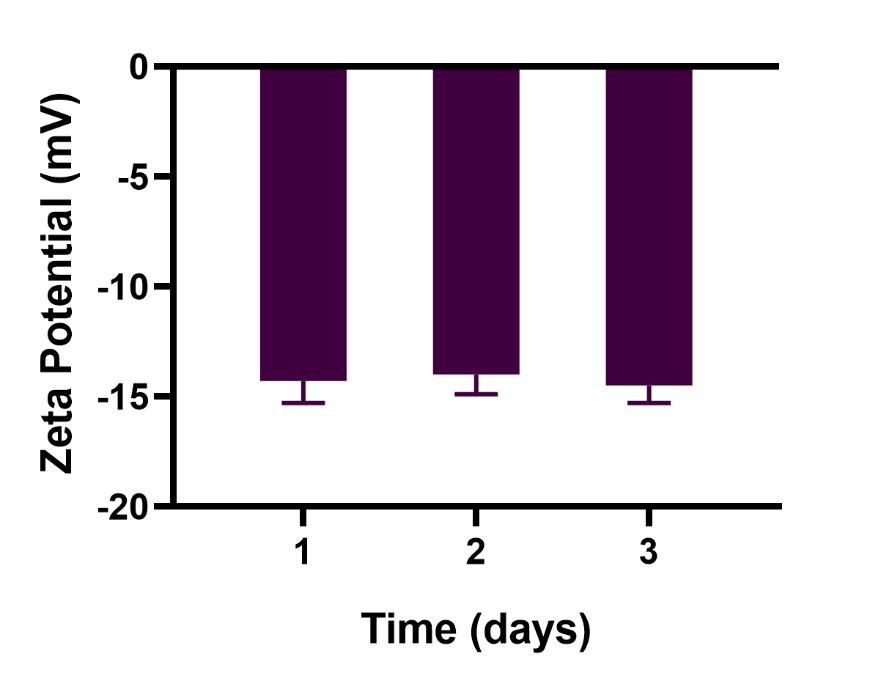


Figure S1. The zeta potential of FeS_2_ suspended in PBS was assessed after 1, 2, and 3 days.


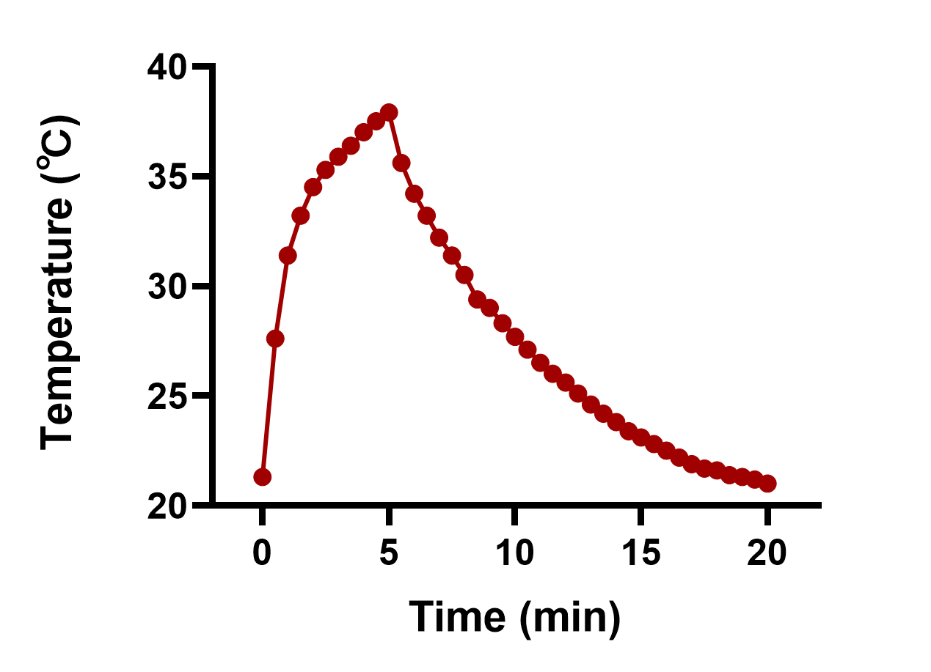


Figure S2. Temperature profile of a FeS_2_ solution under heating with the laser on and then cooling with the laser off.


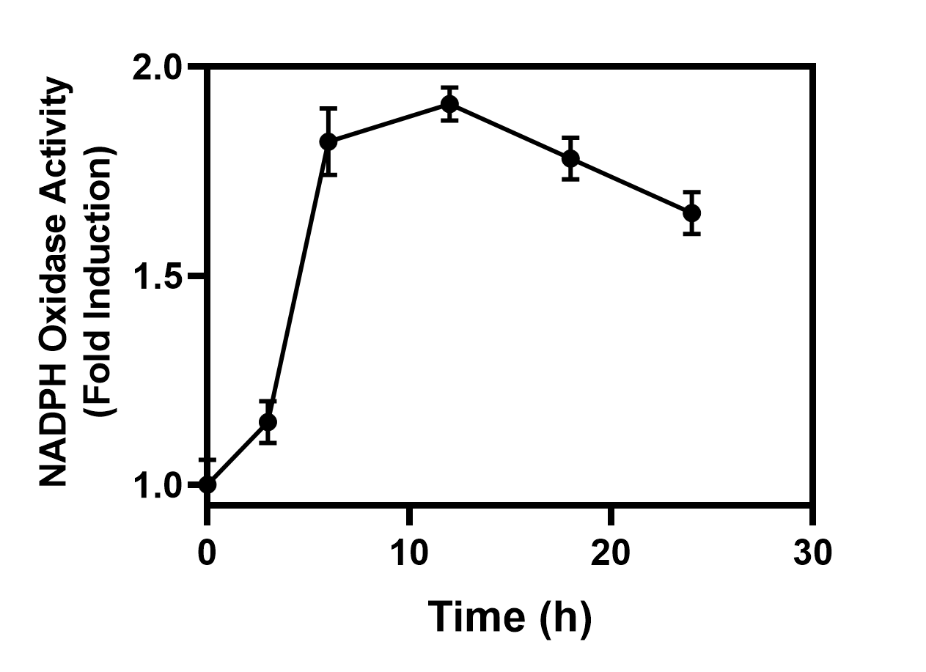


Figure S3. The activity analysis of NADPH oxidase (NOX) in 4T1 cells after treated with 10 μg/mL CPT for different time points.


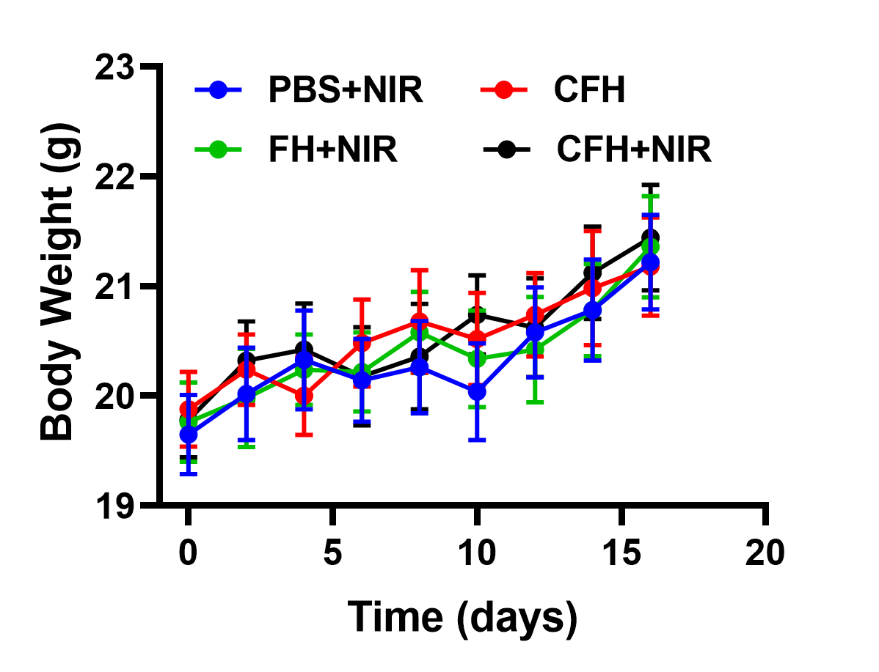


Figure S4. Body weight recorded after different treatments.

[1] C. Huang, Z. Liu, M. Chen, L. Du, C. Liu, S. Wang, Y. Zheng, W. Liu, Tumor-derived biomimetic nanozyme with immune evasion ability for synergistically enhanced low dose radiotherapy, Journal of nanobiotechnology 19(1) (2021) 457.

[2] D. Zhu, M. Lyu, Q. Huang, M. Suo, Y. Liu, W. Jiang, Y. Duo, K. Fan, Stellate Plasmonic Exosomes for Penetrative Targeting Tumor NIR-II Thermo-Radiotherapy, ACS applied materials & interfaces 12(33) (2020) 36928-36937.
